# Using Baidu index to nowcast hand-foot-mouth disease in China: a meta learning approach

**DOI:** 10.1186/s12879-018-3285-4

**Published:** 2018-08-13

**Authors:** Yang Zhao, Qinneng Xu, Yupeng Chen, Kwok Leung Tsui

**Affiliations:** 1 Centre for System Informatics Engineering, City University of Hong Kong, Tat Chee Avenue, Kowloon, Hong Kong Special Administrative Region, People’s Republic of China; 2 Department of Systems Engineering and Engineering Management, City University of Hong Kong, Tat Chee Avenue, Kowloon, Hong Kong Special Administrative Region, People’s Republic of China

**Keywords:** HFMD, Baidu index, Predictive model, Meta-learning

## Abstract

**Background:**

Hand, foot, and mouth disease (HFMD) has been recognized as one of the leading infectious diseases among children in China, which causes hundreds of annual deaths since 2008. In China, the reports of monthly HFMD cases usually have a delay of 1–2 months due to the time needed for collecting and processing clinical information. This time lag is far from optimal for policymakers making decisions. To alleviate this information gap, this study uses a meta learning framework and combines publicly Internet-based information (Baidu search queries) for real-time estimation of HFMD cases.

**Methods:**

We incorporate Baidu index into modeling to nowcast the monthly HFMD incidences in Guangxi, Zhejiang, Henan provinces and the whole China. We develop a meta learning framework to select appropriate predictive model based on the statistical and time series meta features. Our proposed approach is assessed for the HFMD cases within the time period from July 2015 to June 2016 using multiple evaluation metrics including root mean squared error (RMSE) and correlation coefficient (Corr).

**Results:**

For the four areas: whole China, Guangxi, Zhejiang, and Henan, our approach is superior to the best competing models, reducing the RMSE by 37, 20, 20, and 30% respectively. Compared with all the alternative predictive methods, our estimates show the strongest correlation with the observations.

**Conclusions:**

In this study, the proposed meta learning method significantly improves the HFMD prediction accuracy, demonstrating that: (1) the Internet-based information offers the possibility for effective HFMD nowcasts; (2) the meta learning approach is capable of adapting to a wide variety of data, and enables selecting appropriate method for improving the nowcasting accuracy.

**Electronic supplementary material:**

The online version of this article (10.1186/s12879-018-3285-4) contains supplementary material, which is available to authorized users.

## Background

Hand, foot and mouth disease (HFMD), usually caused by enterovirus 71 (EV71) and coxsackievirus A16 (Cox a16), is a type of infectious disease that occurs most commonly among children under 5 years old [1–4]. The typical symptoms of HFMD patients include fever, skin eruptions on hands and feet, and vesicles in the mouth. HFMD can cause mild to severe illness. Some patients, especially those infected by EV71, would rapidly deteriorate with life-threatening neurological and systemic complications, including neurological, cardiovascular and respiratory problems. Several large outbreaks of HFMD have been witnessed in Asia-Pacific region in recent decades, such as the 1997 pandemic in Malaysia, 1998 pandemic in Taiwan, 2000 pandemic in Japan, 2008 pandemic in Singapore, Vietnam, Mongolia and Brunei, 2008 to 2012 pandemics in China, 2011 pandemic in Japan, 2012 pandemic in Cambodia and 2015 pandemic in Syria [5–11], posing a heavy burden to public health and socioeconomic system in the affected areas [12]. HFMD has been recognized as one of the leading infectious diseases among children in China, which causes hundreds of annual deaths since 2008 [4, 13]. Real-time epidemiological surveillance and early warning of HFMD could enable the timely interventions to prevent and control HFMD outbreaks, thus effectively minimizing morbidity, mortality, and reducing the cost of public health system.

China has built its surveillance system to report the monthly HFMD cases and mortality, but the report always has a 1–2 months delay which could be a major challenge for policymakers to accurately estimate epidemics in an efficient real-time manner. Therefore, an effective system that enables forecasting current HFMD (i.e. nowcasting) is in urgent need. An up-to-date detection of acute disease outbreak means more days gained, more lives and more resources saved. In the previous studies, various time series models have been employed for HFMD prediction based on historical reports, including autoregressive integrated moving average (ARIMA) and season ARIMA (SARIMA) [14–18]. However, ARIMA based models have a disadvantage in common that they are essentially ’backward-looking’, which results in poor prediction at turning points unless the turning point represents a return to a long-run equilibrium [19]. Several studies discovered the correlation between the trend of HFMD and some external variables, where the prediction models are constructed by incorporating external variables such as meteorological data and calendar variables [20–27]. However, one limitation of those models is that they can only be used in a relatively small area, such as a town, and may not be applicable in larger areas due to geographical variety of those external variables among sub-areas. Thus, how to predict HFMD epidemics effectively in larger scales, such as in a province or entire China, remains an open question for researchers.

With the arrival of big data era, we are encountering large streaming data in our lives more frequently than ever before. The availability of big data from multiple sources provides new opportunities and tools for evident-supported decision making, such as infectious diseases prediction. In 2008, Google developed an influenza surveillance web-service, namely GFT (Google Flu Trends) [28], which used the Google search query as external variables to predict weekly influenza-likeliness (ILI) rate. The success of GFT motivated several studies aiming to assess current flu activity based on secondary data such as Internet search queries and electronic health records [29–35]. Several studies have been conducted on HFMD prediction using Baidu search queries [36–38]. In these research works, Baidu search queries are incorporated into forecasting methods, and the HFMD prediction is either at provincial or national level. In fact, both data-driven and knowledge-driven forecasting methods usually work well in specific conditions, which is due to the inherent diversities among data sets. The forecasting accuracy can be completely varied when there exists some difference in data structure, data size, time scale, etc. [39, 40]. Therefore, how to develop a robust method or framework with effective model selection for epidemics prediction is a major concern for many applications of public health surveillance.

Our contribution in this paper is two folds: (1) We comprehensively investigate the predictive utility of search queries from Baidu, a dominating search engine in China, for predicting the number of HFMD cases in China, and (2) We develop a novel meta learning (ML) framework that incorporates Internet big data and various parametric predictive models for improving the nowcasting accuracy of HFMD. We evaluate the prediction performance of our estimates in terms of root mean squared error (RMSE) and correlation coefficients (Corr). The results show that: the prediction performance of the predictive models and methods can be significantly improved by utilizing Internet-based search data; the developed meta learning approach can automatically select befitting model based on the historical information, and is more efficient than using single model in terms of prediction power.

## Methods

### Data source and process

In this study, we focus on the problem of nowcasting monthly HFMD cases in areas with geographical variety including Guangxi province, Zhejiang province, Henan province, and China. The reason that we choose these provinces is that most HFMD cases occur in central and southern China [13]. The surveillance data in China, Guangxi, and Zhejiang cover four years from July 2012 to June 2016, and the data in Henan are from January 2013 to June 2016. We collect the monthly reported clinical cases of HFMD from Chinese Centers for Disease Control and Prevention (CDC) and CDCs in the specific provinces accordingly. In medical informatics, an HFMD case is defined as having clinical confirmation of popular vesicular rashes on hands, feet, mouth or buttocks, with or without fever [4].

**Fig. 1:**
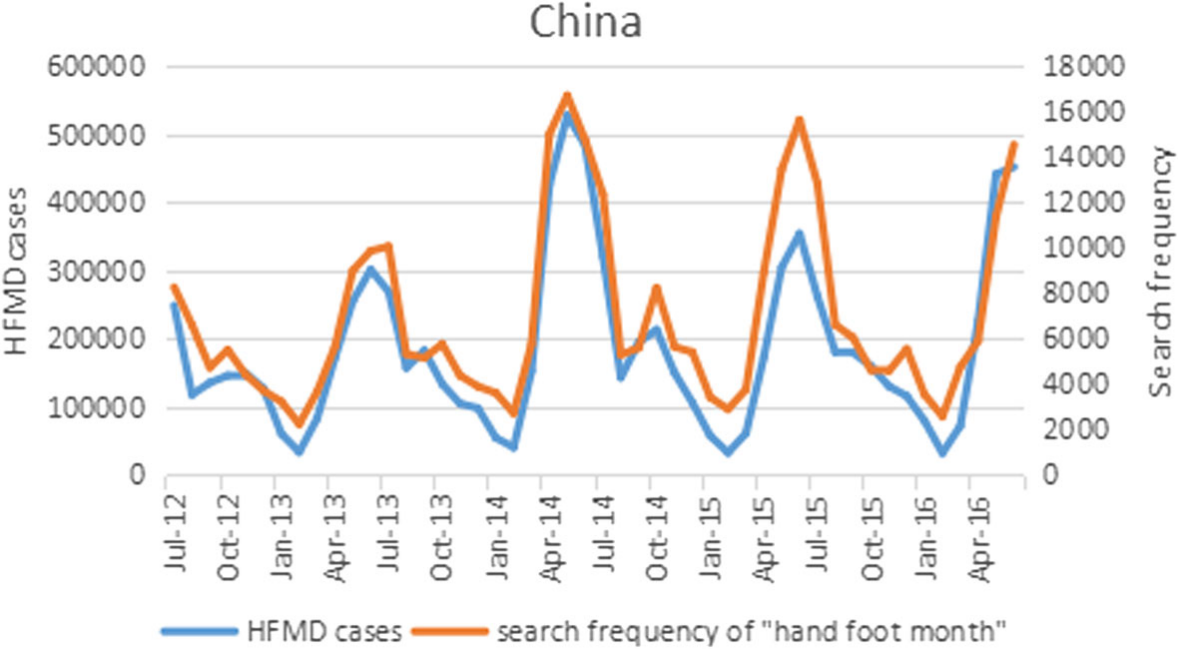
Monthly HFMD cases in China and search frequency of ‘hand-foot-mouth’. Blue: the variation trend of monthly HFMD incidences in China; Orange: Baidu search volume of ‘hand-footmouth’

Baidu is the most prevailing Web search engine in China with over 80 percent of market share [41]. Among the various online services provided by Baidu, Baidu Index (https://index.baidu.com) is an online search tool that allows users to view how frequent the specific keywords, subjects and phrases have been queried over a time period. In this study, we use the HFMD related search frequency of keywords obtained from Baidu Index as external variables to predict HFMD epidemics. We select search terms or keywords which are closely correlated with HFMD epidemics from a keyword tool ’Chinaz’ (http://tool.chinaz.com) [12]. The keywords are obtained through calculating their pairwise correlation with HFMD time series data, using semantic correlation analysis on the relevant queries in Baidu from any available portal websites, blogs, and online reports. Finally, 46 top keywords are selected as the most correlated to the China HFMD cases (the selected Chinese keywords are displayed in the Additional file 1: Table S1). We collect daily search query of these keywords via Baidu Index, and then aggregate the data to a monthly basis for consistency. Figure 1 illustrates the HFMD associated queries, where the monthly HMFD cases in China and search frequency of Chinese keyword ‘hand-foot-mouth’ are plotted for comparison. As can be seen in Fig. 1, the two time series are highly correlated.

### Study design

In our case, the response variable is the monthly HFMD incidences and the covariates are the Baidu index of the selected search keywords. The correlation coefficients are calculated, and only those search terms whose correlation coefficients are higher than 0.5 are used in the subsequent predictive models. The keywords used thus might be different for predicting the HFMD cases in each month. Our proposed approach also employs autoregressive terms because of the intrinsic time series structure in HFMD observations. Let *y*_*i*_ denote the number of HFMD cases in month *i*, we calculate the correlation coefficients between the HFMD observations at lag 0 (*y*_*i*_) and observations at lag 1, 2, 3, 4, 5, and 6 (*y*_*i*−1_,…,*y*_*i*−6_), respectively. As can be seen in Table 1, the HFMD cases at lag 1 is significantly associated with the current HFMD incidence in terms of correlation coefficients. The autoregressive term *y*_*i*−1_ together with the Baidu index of search keywords comprise the covariates in our proposed approach.

**Table 1:** Correlation coefficients of HFMD cases at lag 0 with cases at lag 1, 2, 3, 4, 5, and 6

Region	Lag 1	Lag 2	Lag 3	Lag 4	Lag 5	Lag 6
China	0.744	0.236	-0.194	-0.398	-0.387	-0.368
Guangxi	0.667	0.089	-0.241	-0.281	-0.206	-0.125
Henan	0.714	0.198	-0.152	-0.298	-0.324	-0.33
Zhejiang	0.675	0.197	-0.106	-0.211	-0.083	-0.039

It is observed that the number of covariates exceeds the number of cases in our data sets, least squares estimation may be ill-posed when using linear regression [42]. Three methods, including principal component analysis (PCA), least absolute shrinkage and selection operator (LASSO), and ridge regression (RR), are employed in our model to tackle this problem. In addition, we use autoregressive integrated moving average (ARIMA) to predict the incidence of HFMD in four regions, because of the underlying time series structure of HFMD observations.

Since the relationship between HFMD cases and Baidu index is intrinsically dynamic we adopt an adaptive form of out-of-sample forecasting in this study [43]. For PCA, LASSO, RR, and ARIMA, we use a 24 months window (i.e. two full years) to train statistical models and then the upcoming months to perform out-of-sample prediction validation. As the available data is limited, the selected 24 months window length can also capture the yearly trend as well as seasonal pattern. The model parameters are recomputed before predicting each point by using the training data from the previous 24 months.

### Evaluation metrics

Three metrics are employed to measure the prediction accuracy: root mean square error (RMSE), mean absolute percent error (MAPE) and correlation coefficient (Corr). For a series of predicted values $\hat {\boldsymbol {Y}}=(\hat {y}_{1}, \hat {y}_{2}, \ldots, \hat {y}_{n})$ and their corresponding real values ***Y***=(*y*_1_,*y*_2_,…,*y*_*n*_), these metrics are

$$\begin{array}{@{}rcl@{}} RMSE&=&\sqrt{\frac{\sum_{i=1}^{n}\left(\hat{y}_{i}-y_{i}\right)^{2}}{n}},\\ MAPE&=&\frac{\sum_{i=1}^{n}\left(\left|\frac{\hat{y}_{i}-y_{i}}{y_{i}}\right|\right)}{n}\\ Corr&=&\frac{cov\left(\hat{\boldsymbol{Y}},\boldsymbol{Y}\right)}{\sigma_{\hat{\boldsymbol{Y}}} \sigma_{\boldsymbol{Y}}}. \end{array} $$

Smaller RMSE and MAPE indicates the better prediction performance, while the higher the correlation the better.

### Statistical methods

#### A meta learning approach for HFMD nowcasting

As discussed earlier, one major challenge of health forecasting is that there is no single algorithm performs best for all health conditions. Although four individual models are examined in this study, there is no guarantee that one of them can always outperform the others. To achieve more accurate forecasting result, an important question is how to choose the best model for each time point in each location. Meta-learning approach, in this scenario, is a potential approach to automatically acquire empirical knowledge for supporting non-expert users in algorithm selection task [44]. Meta-learning has proven to be effective in many forecasting applications [45–48], but its effectiveness in forecasting infectious diseases has been rarely investigated.

Meta-learning is defined as an automatic process of generating knowledge associating the performance of algorithms to the characteristics of problem [49]. The meta learner can simply be a single machine learning algorithm [50]. In this case, we employ support vector machine (SVM) as the meta learner to build the recommendation system in meta learning. SVM is a specific class of algorithms, characterized by the usage of kernels, absence of local minima, sparseness of the solution and number of support vectors, etc [51]. SVM can be applied for both classification and regression purpose. In SVM classification, the goal is to find a maximal margin hyper-plane that separates data points from different classes as wide as possible in feature space. Besides linear classification, SVMs also works efficiently in cases of nonlinear separation via kernel transformation, which can automatically map their inputs into the transformed feature spaces.

**Fig. 2:**
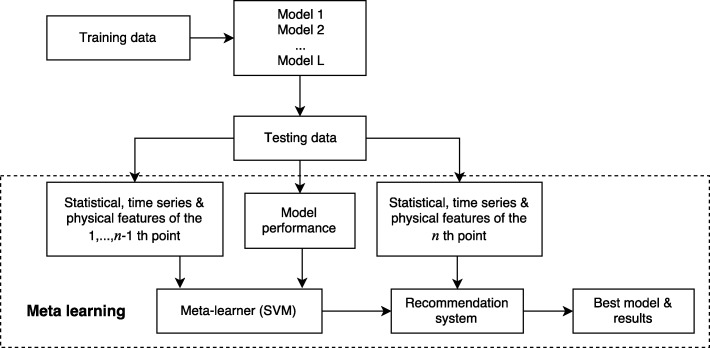
Meta learning framework

Figure 2 shows the overall procedure of our meta learning framework. We take the HFMD forecasting in China as an example to illustrate the framework. Let ***Y*** = (*y*_1_…*y*_48_)^⊤^ represent the outputs, where *y*_*i*_(*i* = 1,…,48) denotes the monthly HFMD incidences in China from July 2012 to June 2016. Let ***X***=(***x***_1_…***x***_48_)^⊤^ represent the covariates set, where ***x***_*i*_ = (1,*y*_*i*−1_,***b***_*i*_) denotes the *i*th input, and ***b***_*i*_=(*b*_*i*1_,…,*b*_*ik*_) denotes the Baidu index (search frequency) of *k* (*k*=46) search keywords related to HFMD activity in the *i*th month. The procedure of meta learning method mainly consists of the following steps:

Step 1: The dataset is divided into training set ***T***^(0)^ and testing set ***T***^(1)^. For training set ***T***^(0)^, $\boldsymbol {t}_{j}^{(0)}=\left (y_{j},\boldsymbol {x}_{j}\right) (j=1,\ldots,26)$ is the *j*th point of training set, where ***x***_*j*_=(*y*_*j*−1_,***b***_*j*_). For testing set ***T***^(1)^, $\boldsymbol {t}_{s}^{(1)}=\left (y_{s},\boldsymbol {x}_{s}\right) (s=1,\ldots,22)$ is the *s*th point of testing set, where ***x***_*s*_=(*y*_*s*−1_,***b***_*s*_).

Step 2: A set of predictive method candidates {*f*^(1)^,…,*f*^(*L*)^} for fitting the relationship between ***Y*** and ***X*** is constructed. For each method, we have the fitted model *y*_*i*_=*f*^(*l*)^(***x***_*i*_;***θ***^(*l*)^), where *f*^(*l*)^∈{*f*^(1)^,…,*f*^(*L*)^} and ***θ***^(*l*)^ is the parameter set of this method. For each data point in testing set, all the predictive methods are applied for HFMD prediction and an adaptive approach (models are dynamically trained with a 2-year time window) is adopted.

Step 3: The MAPE of each predictive method at the first *n*−1 testing data points is calculated, and the optimal method is selected by minimizing MAPE value, i.e. $l_{s}^{*}=arg\min \limits _{l\in \{1,\ldots,L\}}{MAPE}_{s}=arg\min \limits _{l\in \{1,\ldots,L\}}\left |\hat {y}_{s}^{(l)}-y_{s}^{(l)}\right |/y_{s}^{(l)}$;

Step 4: For each case in the first *n*−1 testing data points, 11 statistical, time series and physical features characterizing its training set are extracted based on previous study [46–48, 50]. Let $\boldsymbol {F}_{s}=\left (F_{s}^{1},\ldots,F_{s}^{m}\right)$ denote the set of features. The description of the features is shown in Table 2.

**Table 2:** Meta features description

Feature	Explanation
Min	Minimum of the HFMD cases over the time period.
Max	Maximum of the HFMD cases over the time period.
Mean	Mean of the HFMD cases over the time period.
SD	Standard deviation of the HFMD cases over the time period.
SKEW	Skewness of the HFMD cases over the time period.
KURT	Kurtosis of the HFMD cases over the time period.
Q1	First quartile of the HFMD cases over the time period.
Q2	Second quartile of the HFMD cases over the time period.
Q3	Third quartile of the HFMD cases over the time period.
Month	Calendar month of the forecast point.
Ratio of turning points	Percentage of turning points in the series.

Step 5: SVM is employed as the meta leaner to train the data set $\left (l_{s}^{*},\boldsymbol {F}_{s}\right)(s=1,\ldots,n-1)$, where $l_{s}^{*}$ is the response variable, which is the optimal method index for the *s*th point, and the 11 features ***F***_*s*_ extracted from the corresponding training set are the covariates. Leave-One-Out Cross Validation is applied for model parameter tuning. The fitted model will be sent to the recommendation system for selecting optimal method on a given data set.

Step 6: To predict new HFMD cases in the *n*th month, the 11 features associated with its training set will be input to the recommendation system, then the meta learner will return an appropriate method for forecasting HFMD incidences in the *n*th month. The new HFMD cases will be predicted via the recommended model.

#### Linear regression (LR) with principle component analysis (PCA)

Linear regression (LR) was the first type of regression method with complete theoretical system, and to be applied widely in practical applications. In this study, the linear regression model is formulated as:

$$\begin{array}{@{}rcl@{}} y_{i}=\alpha+\beta_{0}y_{i-1}+\sum_{k=1}^{46}{\beta_{k}b_{ik}}+\varepsilon_{i}, \qquad \varepsilon_{i}\overset{iid}{\sim}N\left(0, \sigma^{2}\right) \end{array} $$

Letting $\hat {\boldsymbol {\beta }}=\left (\hat {\beta }_{0}\ldots \hat {\beta }_{46}\right)$, where ***b***_*i*_ are the exogenous variables.

However, as mentioned earlier, LR might be ill-posed when the number of covariates exceeds the number of cases due to the limitation of least squares estimation. To tackle this problem, we introduce Principal Component Analysis (PCA) to reduce the dimensionality of covariates. PCA works by first computing linear combinations of variables that contribute to variation in the sample, and then ranking the combinations of variables according to the amount of variations they account for. The most contributed combinations of variables are then used as the new covariates for regression. More details of application of PCA can be referred to [52–56]. In this study, we apply PCA on the observed Baidu index matrix of training set to obtain the principal components, and select a subset of the top principal components that explain at least 95% variance.

#### Least absolute shrinkage and selection operator (LASSO)

LASSO, which is referred to as L1 regularization method, is able to achieve both covariates selection and regression. It works by setting a constraint on the sum of the absolute value of the regression coefficients, forcing certain coefficients to be zeros. In this way, LASSO enables efficient selection of a simpler model without the insignificant features, which could enhance predication accuracy. More technical details of LASSO and its some generalizations and variants can be found in [57, 58]. In this study, the LASSO estimate $(\hat {\alpha },\hat {\boldsymbol {\beta }})_{lasso}$ can be obtained by solving

$$\begin{array}{@{}rcl@{}} \left(\!\hat{\alpha},\hat{\boldsymbol{\beta}}\!\right)_{lasso}&=& arg \min{\sum_{i}\left(y_{i}-\alpha-\beta_{0}y_{i-1}-\!\sum_{j=1}^{46}\beta_{k}b_{ik}\right)^{2}}\\ &&\,subject\ to \sum_{k=0}^{46}\left|\beta_{k}\right|\le g, \end{array} $$

where *g*≥0 is a tuning parameter.

#### Ridge regression (RR)

Ridge regression, which is referred to as L2 regularization method, is also applied for HFMD nowcasting in this study. Ridge regression conducts the least squares estimation by adding a small constant value *λ* to the diagonal entries of the matrix ***X***^*T*^***X*** before taking its inverse. The ridge regression estimate $\left (\hat {\alpha },\hat {\boldsymbol {\beta }}\right)_{ridge}$ can be obtained by solving

$$\begin{array}{@{}rcl@{}} \begin{aligned} \left(\!\hat{\alpha},\hat{\boldsymbol{\beta}}\right)_{ridge}=arg \min{\sum_{i}\left(y_{i}-\alpha-\beta_{0}y_{i-1}-\sum_{j=1}^{46}\beta_{k}b_{ik}\right)^{2}}+\lambda\sum_{k=0}^{n}\beta_{k}^{2} \end{aligned} \end{array} $$

The analytical solution of the ridge regression estimator is given by

$$\begin{array}{@{}rcl@{}} \left(\hat{\alpha},\hat{\boldsymbol{\beta}}\right)_{ridge}=\left(\boldsymbol{X}^{T}\boldsymbol{X}+\lambda\boldsymbol{I}\right)^{-1}\boldsymbol{X}^{T}\boldsymbol{y}, \end{array} $$

where ***I*** is an identity matrix.

Different from LASSO, ridge regression is more commonly used to deal with the collinearity among variables. More details of ridge regression and its applications can be found in [59–61].

#### Autoregressive integrated moving average (ARIMA)

Besides regression-based approaches, we also consider autoregressive integrated moving average ARIMA(*p*,*d*,*q*) model, where *p* is the number of autoregressive (AR) terms, *q* is the order of the non-seasonal moving average (MA) lags, and *d* is the number of non-seasonal differences [62–64]. ARIMA model can be formulated as:

$$\begin{array}{@{}rcl@{}} y_{t}=\vartheta_{0}+\sum_{i=1}^{p}{\varphi_{i}y_{t-i}}+\sum_{j=1}^{q}{\vartheta_{j}\varepsilon_{t-j}^{arima}}+\varepsilon_{t+h}^{arima}, \end{array} $$

where *y*_*t*_ is the number of HFMD cases at time t and $\varepsilon _{t}^{arima}$ is white noise random error; *φ*_*i*_ (*i* = 1,2,…,*p*) and *𝜗*_*j*_ (*j* = 0,1,2,…,*q*) are parameters to be estimated via least squares or maximum likelihood estimation. The parameters *p*, *q*, and *d* are selected from a search over all the possible model candidates by minimizing the corrected Akaike Information Criterion (AIC) [65].

Time series models can provide satisfactory forecasting performance when the time series data have clear trend and seasonality. However, the strong assumption of the statistical properties of time series data might limit the reliability of forecast performance.

All of the experiments are implemented in R v3.4.1(64 bit) platform using the “MASS”, “penalized”, “hydroGOF”, “forecast”, “glmnet”, “moments”, “e1071”, and “kernalb” packages [66].

## Results

We evaluate and compare the forecasting performance of each method. For the time period from July 2015 to June 2016, the meta learning approach reduces the RMSE of the compared method which has the minimum RMSE by 37%, 20%, 20%, and 30% for the four regions, i.e. China, Guangxi, Zhejiang, and Henan, respectively. Comparing the correlation between the nowcasting results and observations, the prediction of the meta learning approach has the maximum correlation coefficient with the ground truth.

**Fig. 3:**
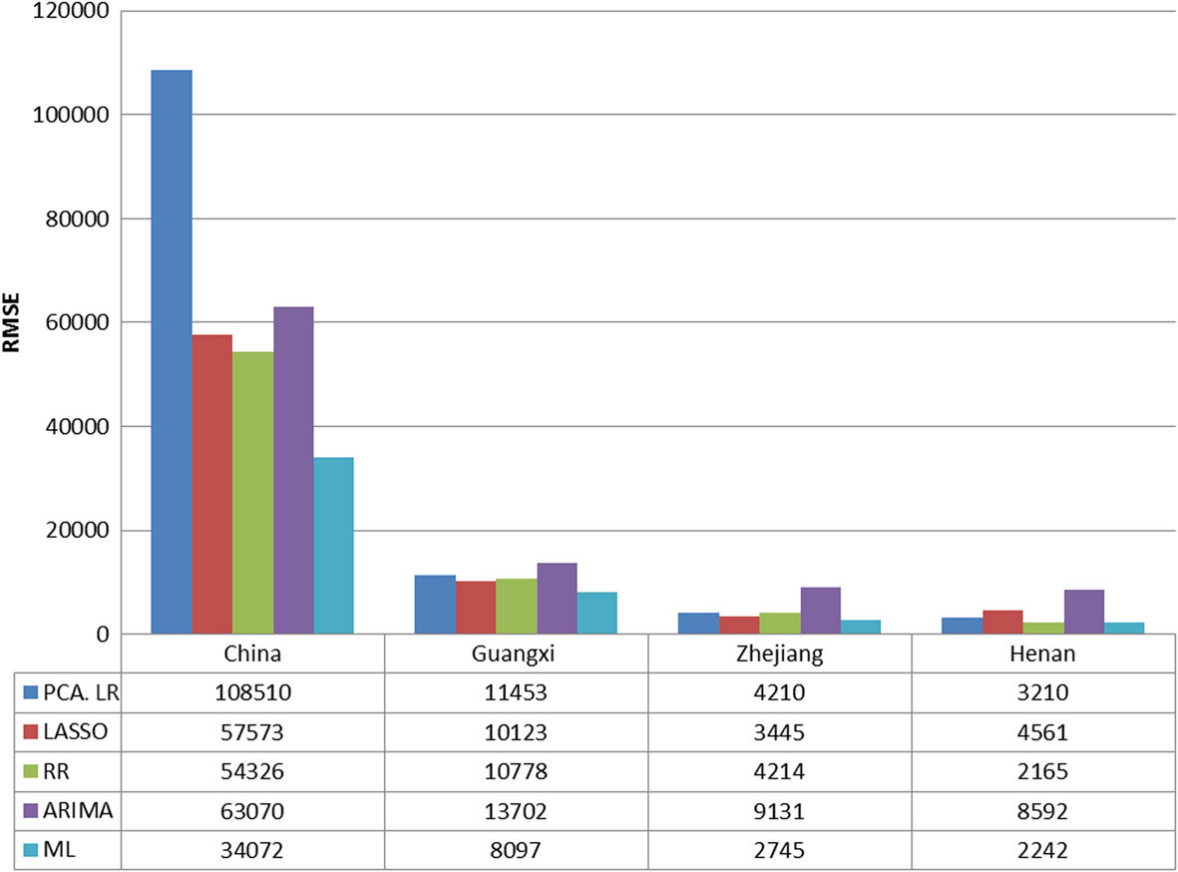
Evaluation metric: RMSE. Dark blue: the RMSE of PCA; Red: the RMSE of LASSO; Green: the RMSE of RR; Purple: the RMSE of ARIMA; Light blue: the RMSE of ML

**Fig. 4:**
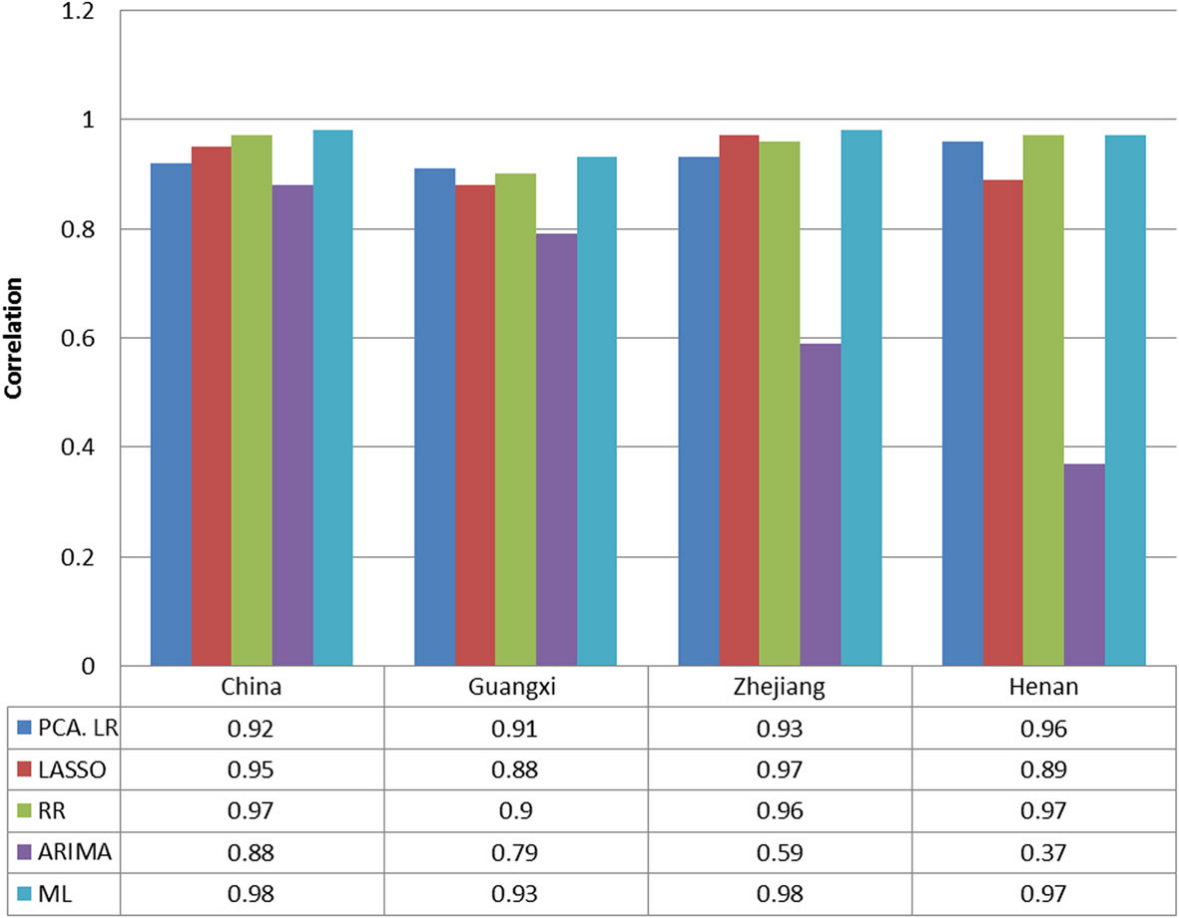
Evaluation metric: correlation coefficient. Dark blue: the correlation coefficient of PCA; Red: the correlation coefficient of LASSO; Green: the correlation coefficient of RR; Purple: the correlation coefficient of ARIMA; Light blue: the correlation coefficient of ML

Figures 3 and 4 show the RMSE and correlation coefficient of the compared predictive methods in different regions, respectively. As can be seen from the figures, the result verifies the fact that no single model outperforms other models in the four regions. PCA shows inconsistent forecasting performance as it performs worst in China and is comparable with RR and LASSO in the three provinces. The two regularization methods, i.e. LASSO and RR, are competitive in most of the cases except in Henan, where PCA outperforms LASSO. ARIMA does not perform well in all the four regions compared with the models with Baidu index, especially in the three provinces where it is always the worst among the four individual models, validating the predictive utility of Baidu search queries. Comparing the proposed meta learning approach with each individual model, it performs best in China, Guangxi, and Zhejiang, while it is as good as RR in Henan, indicating the effectiveness of meta learning in selecting the befitting models.

**Fig. 5:**
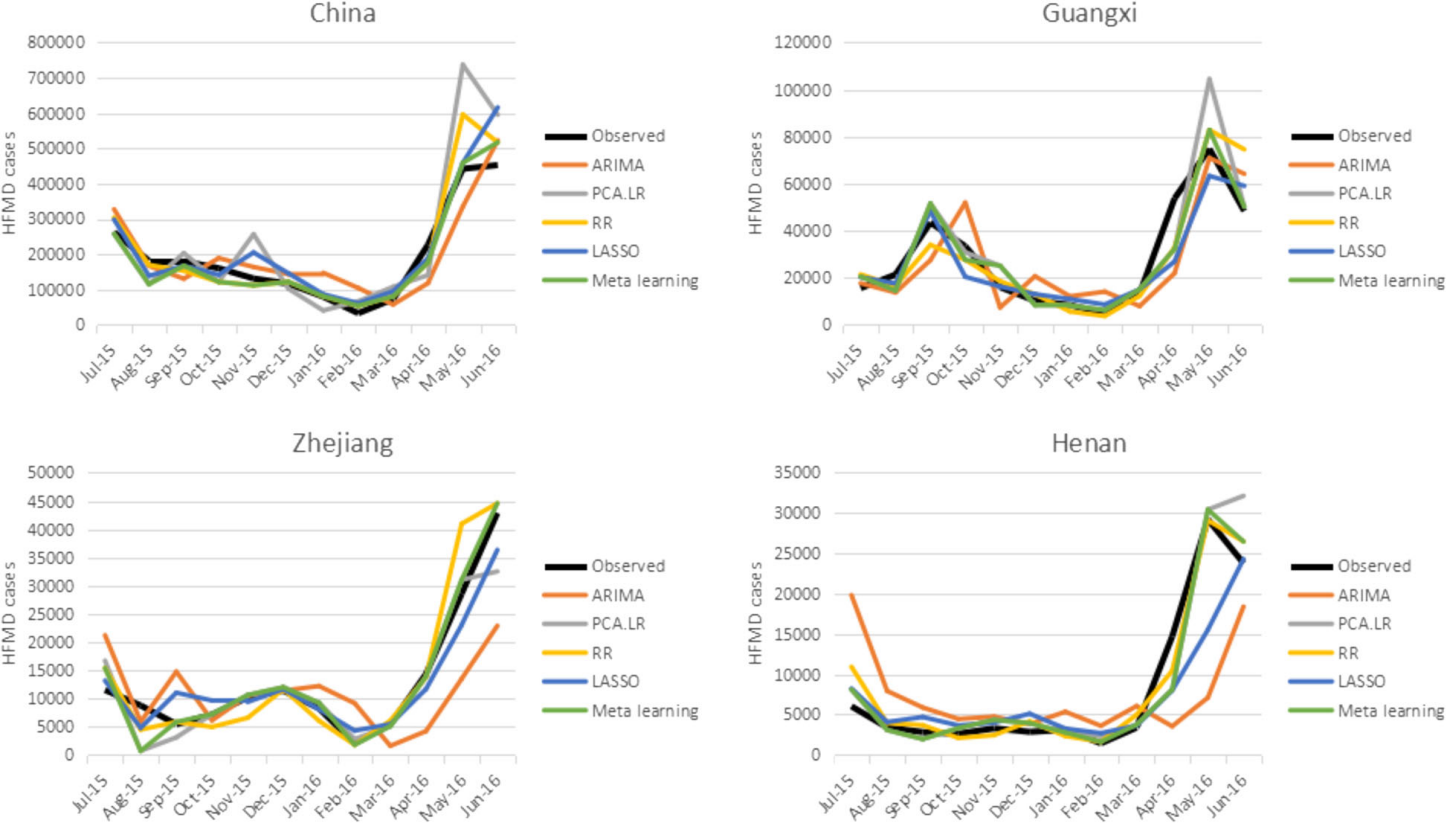
Forecasting results. Black: the true value; Orangered: the nowcasting results of ARIMA; Gray: the nowcasting results of PCA; Orange: the nowcasting results of RR; Dark blue: the nowcasting results of LASSO; Green: the nowcasting results of Meta learning

The comparison of the prediction results over the entire forecasting period of all the methods is displayed in Fig. 5 (The numerical results can be found in Additional file 2: Table S2). Clearly, ARIMA model shows delayed (or “off”) prediction performances in all the regions, as ARIMA only relies on the historical time series data and it is not able to capture the irregular turning point which contributes to the delayed prediction of those points. PCA, LASSO, and RR can capture the seasonal pattern of HFMD epidemics more accurately, but PCA greatly overestimates the HFMD cases at some time points. At most of the forecasting points, meta learning can match the best or one of the best two models, and there is few significant overestimation or underestimation throughout the forecasting period.

Furthermore, in order to further demonstrate the predictive utility of models incorporating Baidu search queries, we compare regression based models with and without Baidu index data. For models without Baidu index data, the three models including PCA+LR, LASSO, and RR degrade into classical linear regression (LR), as only the HFMD cases at lag 1 (*y*_*t*−1_) is left as covariate. Tables 3 and 4 show the RMSE and Corr of the four compared forecasting models (LR, PCA+LR, LASSO, RR) in different regions. As can be seen from the results, PCA.LR, LASSO and RR (models with Baidu data) shows better predictive performance than LR (model without Baidu data), indicating the utility of Baidu search queries.

**Table 3:** RMSE of different forecasting methods

	Model without Baidu data	Model with Baidu data		
Region	LR	PCA.LR	LASSO	RR
China	150860	108510	57573	54326
Guangxi	13062	11453	10123	10778
Zhejiang	7682	4210	3445	4214
Henan	7028	3210	4561	2165

**Table 4:** Corr of different forecasting methods

	Model without Baidu data	Model with Baidu data		
Region	LR	PCA.LR	LASSO	RR
China	0.74	0.92	0.95	0.97
Guangxi	0.65	0.91	0.88	0.90
Zhejiang	0.64	0.93	0.97	0.96
Henan	0.65	0.96	0.89	0.97

## Discussion

In this paper, we evaluated the predictive utility of Baidu search data in nowcasting HFMD cases in China. The conventional linear regression is not appropriate for this problem due to the relatively large number of covariates in the model. Therefore, we employ four parametric models, including PCA, RR, LASSO, and ARIMA, to nowcast monthly HFMD cases in China, Guangxi province, Zhejiang province, and Henan province.

The result suggests that the time series model, ARIMA, underperforms due to its delayed prediction performance. PCA, LASSO, and RR have the competitive performances in most of the regions and produce more accurate prediction than ARIMA. Among the compared methods, PCA overestimates or underestimates the HFMD epidemics at some forecasting points, and performs slightly worse than LASSO and RR. The performance of LASSO and RR are similar.

In general, PCA, LASSO, and RR can be feasible single model to nowcast HFMD cases in province or country scales by using Baidu search data when there are limited observations and a relatively large number of search terms. However, they could not produce consistently accurate HFMD nowcasting results because of the relatively weak robustness of each model. No single predictive method proves to be universally best in the four cases.

This result motivates us to develop a novel model selection approach in order to choose appropriate model in different situations. The meta learning approach is then developed to fulfill the requirement. Specifically, the meta learning framework consists of a two-stage learning process: In Stage 1, the features characterizing the problem are extracted based on historical data; In Stage 2, a meta learner module is built to learn the interrelation between the features and model performances from the known facts, and deduce new knowledge and rules. This meta learning approach with automatic model recommendation system is superior to the compared individual methods in the problem of HFMD nowcasting.

**Fig. 6:**
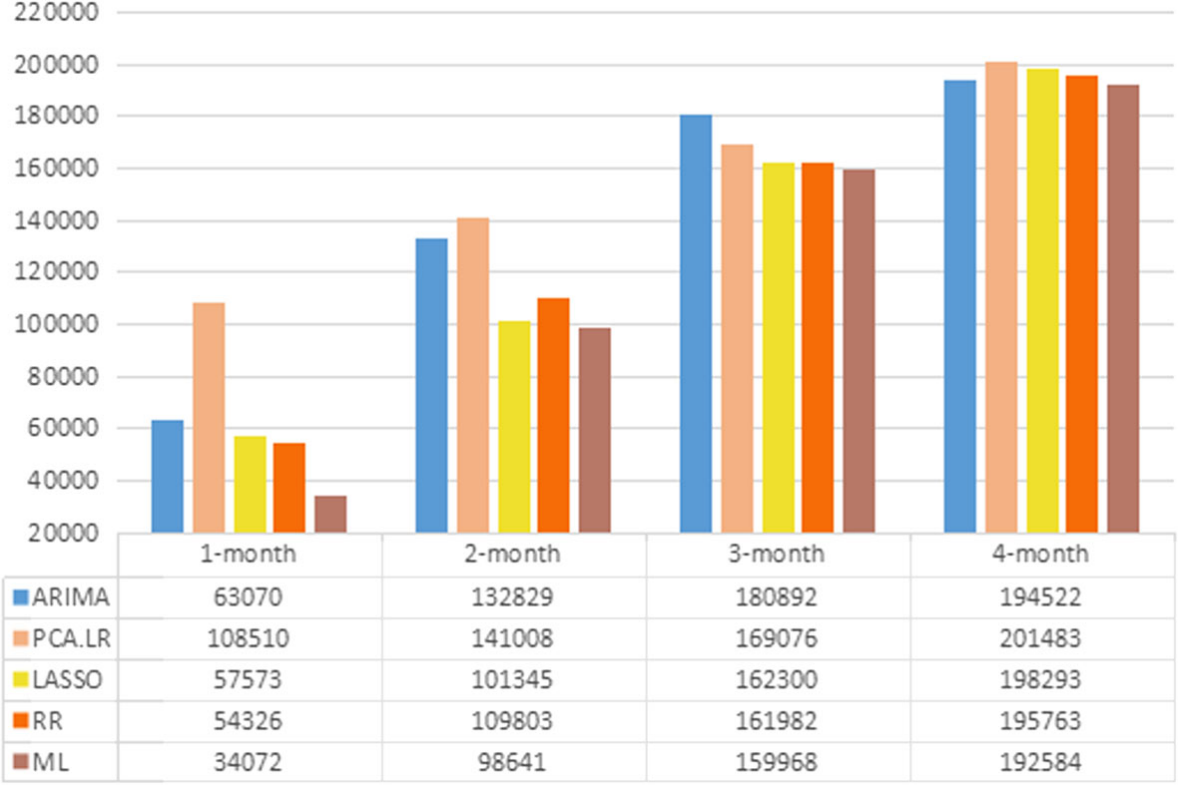
Evaluation metric of different lag time: RMSE. Blue: the RMSE of ARIMA; Orange: the RMSE of PCA+LR; Yellow: the RMSE of LASSO; Orangered: the RMSE of RR; Brown: the RMSE of ML

In this paper, we focus on HFMD nowcasting with 1-month lag data. It should be noted that the prediction power of forecasting method may degrade as time lag increases. In the following, we take the HFMD nowcasting in the whole China as an example for further illustration. Similar to the 1-month nowcasting, the metric RMSE is used to evaluate the prediction performance of the nowcasting with varied time lag. Figure 6 shows the evaluation results in terms of RMSE of the five compared forecasting methods including meta learning, ARIMA, PCA+LR, LASSO and RR. As can be seen from Fig. 6, the prediction accuracy of various methods declines with the increase of time lag (i.e. from 1 month to 4 months), which is consistent with our findings in the preliminary analysis that the more recent HFMD activities are more associated with the current HFMD incidence in terms of Corr. In spite of the varied time lag, the proposed ML framework still outperforms the other methods, indicating its robustness and effectiveness; as the time lag increases, the difference between the various predictive models’ performance become smaller. It is worth noting that the proposed meta learning approach is not restricted by data resolution, although monthly data is used to illustrate its effectiveness.

The proposed meta learning framework provides practical guidelines in the design, development, implementation, and testing of a forecasting recommendation system for health forecasting problems. Specifically, it can help non-experts with predictive methods selection. One is to further examine the features for meta learner. The meta learning framework can incorporate various predictive methods and machine learning algorithms. In fact, there could be some other effective features than those used in our model, and there are also more choices of machine learning methods for training meta learner, such as deep learning. These will be further investigated in our future work.

## Conclusions

The result of this study demonstrates that the accuracy of HFMD nowcasting can be significantly improved by incorporating Baidu Index data in predictive model. In addition, the developed meta learning approach for model selection together with Baidu Index data enables credible forecasts and provide helpful information for predicting HFMD incidence. Compared with the four individual predictive methods used in this study, the performance of meta learning is more robust for different forecasting scales. Of course, there is still some room for our approach to improve. For example, we will refine the meta learner by examining various learning algorithms in our future work. Besides, we will evaluate the utility of the developed approach in some other forecasting applications.

## Additional files

Additional file 1 **Table S1.** Contains the selected 46 Baidu key words used in predictive models. (PDF 87 kb)

Additional file 2 **Table S2.** Provides the nowcasting results of the monthly HFMD incidences in China, Guangxi, Zhejiang and Henan. (PDF 46 kb)

## Abbreviations

ARIMA: Autoregressive integrated moving average
Corr: Correlation coefficient
HFMD: Hand, foot, and mouth disease
LASSO: Least absolute shrinkage and selection operator
MAPE: Mean absolute percent error
ML: Meta learning
PCA: Principle component analysis
RMSE: Root mean squared error
RR: Ridge regression
