# Supplementary material for: Using Baidu index to nowcast hand-foot-mouth disease in China: a meta learning approach
Source: BMC Infect Dis. 2018 Aug 13;18:398. doi: 10.1186/s12879-018-3285-4 (PMC6090735; doi:10.1186/s12879-018-3285-4)
Supplement: Supplementary file 1 — Table S1. Contains the selected 46 Baidu key words used in predictive models. (PDF 87 kb) [file 12879_2018_3285_MOESM1_ESM.pdf]

Table S1. Baidu search keywords correlated with `HFMD' obtained from `Chinaz'.

No.	Keyword	No.	Keyword	No.	Keyword
1	大人手足口病症<br>symptoms of adult HFMD	2	儿童手足口病<br>children HFMD	3	儿童手足口病症状<br>symptoms of children HFMD
4	如何预防手足口病<br>how to prevent HFMD	5	什么是手足口病<br>what is HFMD	6	手足口<br>hand foot mouth
7	手足口病<br>hand-foot-month-diseases	8	手足口病并发症<br>complication of HFMD	9	手足口病吃什么药<br>what drugs to take to treat HFMD
10	手足口病初期症状图<br>pictures of initial symptoms of HFMD	11	手足口病的传播途径<br>transmission path of HFMD	12	手足口病的预防<br>prevention of HFMD
13	手足口病的症状<br>symptoms of HFMD	14	手足口病的治疗<br>treatment of HFMD	15	手足口病 症状<br>symptoms of HFMD
16	手足口病防治知识<br>knowledge of HFMD prevention and treatment	17	手足口病防治指南<br>guide of HFMD prevention and treatment	18	手足口病疱疹怎么治<br>how to treat herpes of HFMD
19	手足口病前期症状<br>initial symptoms of HFMD	20	手足口病特征<br>features of HFMD	21	手足口病图片<br>pictures of HFMD
22	手足口病严重吗<br>is HFMD severe	23	手足口病疫情<br>HFMD epidemics	24	手足口病应急预案<br>emergency plan of HFMD
25	手足口病用什么药<br>what HFMD drugs to use	26	手足口病用药<br>HFMD drugs	27	手足口病有什么症状<br>what symptoms of HFMD
28	手足口病预防<br>prevention of HFMD	29	手足口病预防控制指南<br>guide of HFMD prevention and control	30	手足口病预防知识<br>knowledge of HFMD prevention
31	手足口病初期症状<br>initial symptoms of HFMD	32	手足口病诊疗指南<br>guide of HFMD treatment	33	手足口病症状<br>symptoms of HFMD
34	手足口病症状图片<br>pictures of symptoms of HFMD	35	手足口病治疗<br>treatment of HFMD	36	手足口病治疗费用<br>treatment charges of HFMD
37	小儿手足口病<br>kids HFMD	38	小儿手足口病的症状<br>symptoms of kids HFMD	39	小儿手足口病症状<br>symptoms of kids HFMD
40	小孩手足口病症状<br>symptoms of children HFMD	41	预防手足口病<br>prevent HFMD	42	怎样预防手足口病<br>how to prevent HFMD
43	重症手足口病<br>severe HFMD	44	手足口病的预防知识<br>knowledge of HFMD prevention	45	手足口病潜伏期<br>incubation of HFMD
46	手足口病症状和治疗<br>symptoms and treatment of HFMD				
