# Supplementary material for: Using Baidu index to nowcast hand-foot-mouth disease in China: a meta learning approach
Source: BMC Infect Dis. 2018 Aug 13;18:398. doi: 10.1186/s12879-018-3285-4 (PMC6090735; doi:10.1186/s12879-018-3285-4)
Supplement: Supplementary file 2 — Table S2. Provides the nowcasting results of the monthly HFMD incidences in China, Guangxi, Zhejiang and Henan. (PDF 46 kb) [file 12879_2018_3285_MOESM2_ESM.pdf]

Table S2.1 Forecasting results of monthly HFMD incidences in China

Date	Observed	ARIMA	PCA.LR	RR	LASSO	Meta learning
Jul-15	262239	327230	257667	303767	298130	257667
Aug-15	180813	166763	114829	169555	138394	114829
Sep-15	180074	130207	203137	152913	167191	167191
Oct-15	160106	188746	121363	121310	141615	121363
Nov-15	130829	163703	256522	111621	204872	111621
Dec-15	116239	143147	101767	121350	145545	121350
Jan-16	79499	147004	40209	80413	86204	80413
Feb-16	32457	104033	66263	53590	60922	53590
Mar-16	72464	57195	107260	80140	94465	80140
Apr-16	226430	117629	140248	175883	187874	175883
May-16	442075	334818	737339	596071	458885	458885
Jun-16	452668	522996	595400	517091	616163	517091

Table S2.2 Forecasting results of monthly HFMD incidences in Guangxi

Date	Observed	ARIMA	PCA.LR	RR	LASSO	Meta learning
Jul-15	15541	17617	20442	21392	20204	20442
Aug-15	21285	13693	14541	16480	17504	14541
Sep-15	43451	27456	51549	33961	48366	51549
Oct-15	33339	51903	30853	27595	20362	27595
Nov-15	15977	7331	25042	18444	16196	25042
Dec-15	10149	20604	8317	12958	12996	8317
Jan-16	8363	12072	8139	5609	10899	8139
Feb-16	4747	14048	6213	3653	8582	6213
Mar-16	13152	7911	15041	12261	15009	15041
Apr-16	53712	21889	32251	32781	26886	32251
May-16	74843	70904	104464	82803	63263	82803
Jun-16	48351	64184	50206	74590	59061	50206

Table S2.3 Forecasting results of monthly HFMD incidences in Zhejiang

Date	Observed	ARIMA	PCA.LR	RR	LASSO	Meta learning
Jul-15	11508	21201	16690	15384	13158	15384
Aug-15	8753	5872	718	4477	4796	718
Sep-15	5446	14746	3110	5843	11023	5843
Oct-15	7140	6122	7202	4961	9644	7202
Nov-15	10197	10497	10608	6566	9376	10608
Dec-15	11408	11395	11996	11634	11666	11996
Jan-16	8254	12205	9230	6046	8077	9230
Feb-16	2060	9157	2810	1787	4299	1787
Mar-16	5419	1600	5095	6064	5439	5095
Apr-16	14306	4204	13909	13775	11663	13909
May-16	28587	13489	31019	40958	23030	31019
Jun-16	42891	22887	32520	44612	36336	44612

Table S2.4 Forecasting results of monthly HFMD incidences in Henan

Date	Observed	ARIMA	PCA.LR	RR	LASSO	Meta learning
Jul-15	6026	19820	8114	10924	8234	8114
Aug-15	3408	7936	3089	3944	4078	3089
Sep-15	2828	5878	1967	3688	4699	1967
Oct-15	2623	4456	3317	2104	3654	3317
Nov-15	3297	4739	4345	2453	3997	4345
Dec-15	2857	3826	3944	4155	5108	3944
Jan-16	3104	5350	2774	2343	3265	2774
Feb-16	1435	3606	2327	1628	2697	1628
Mar-16	3388	6059	3738	4975	3622	3738
Apr-16	14706	3551	8123	10449	8015	8123
May-16	29224	7108	30435	29098	15531	30435
Jun-16	23785	18400	32142	26485	24302	26485
